# Evaluation of Resistance Development in *Bemisia tabaci* Genn. (Homoptera: Aleyrodidae) in Cotton against Different Insecticides

**DOI:** 10.3390/insects12110996

**Published:** 2021-11-05

**Authors:** Muhammad Zaryab Khalid, Sohail Ahmed, Ibrahim Al-Ashkar, Ayman EL Sabagh, Liyun Liu, Guohua Zhong

**Affiliations:** 1 Key Laboratory of Natural Pesticide and Chemical Biology, Ministry of Education, South China Agricultural University, Guangzhou 510642, China; zaryabkhalid0003@hotmail.com; 2 Termite Management Laboratory, Department of Entomology, University of Agriculture Faisalabad, Faisalabad 38000, Pakistan; saha786_pk@yahoo.com; 3 Department of plant production, College of Food and Agriculture, King Saud University, Riyadh 11451, Saudi Arabia; ialashkar@ksu.edu.sa; 4 Department of Agronomy, Faculty of Agriculture, Kafrelsheikh University, Kafr el-Sheikh 33516, Egypt; 5 Graduate School of Integrated Sciences for Life, Hiroshima University, Hiroshima 739-8528, Japan; liuliyun79419@163.com

**Keywords:** *Bemisia tabaci*, cotton, insecticide resistance, pyrethroids, neonicotinoids, chlorfenapyr, buprofezin

## Abstract

**Simple Summary:**

In the tropical and sub-tropical regions of Asia, Africa, and America, the *Bemisia tabaci* (cotton whitefly) has attained a major pest status of cotton. It produces injury to the plant by feeding, excreting honeydews, and by transmitting viruses on many crops. The heavy application of insecticides for controlling the insect pest is one of the main reasons for the outbreaks of whitefly. Due to several reports of control failure of the whitefly, the present study was conducted to evaluate the resistance development in *B. tabaci*. Therefore, the field population of *B. tabaci* was collected, and the resistance development was evaluated against the commonly used insecticides. For evaluating the development of resistance, the *B. tabaci* was selected with the insecticides under the controlled laboratory conditions. The data of mortality was calculated at each generation, and the overall development of resistance up to five generations was evaluated. Results showed that the field collected population was susceptible to the selected insecticides at G1, indicating their effectiveness. However, a continuous selection for only five generations resulted in a significant increase in the resistance development. The present study provided very valuable information on the resistance development in *B. tabaci*.

**Abstract:**

Cotton is a major crop of Pakistan, and *Bemisia tabaci* (Homoptera: Aleyrodidae) is a major pest of cotton. Due to the unwise and indiscriminate use of insecticides, resistance develops more readily in the whitefly. The present study was conducted to evaluate the resistance development in the whitefly against the different insecticides that are still in use. For this purpose, the whitefly population was selected with five concentrations of each insecticide, for five generations. At G1, compared with the laboratory susceptible population, a very low level of resistance was observed against bifenthrin, cypermethrin, acetamiprid, imidacloprid, thiamethoxam, nitenpyram, chlorfenapyr, and buprofezin with a resistance ratio of 3-fold, 2-fold, 1-fold, 4-fold, 3-fold, 3-fold, 3-fold, and 3-fold, respectively. However, the selection for five generations increased the resistance to a very high level against buprofezin (127-fold), and to a high level against imidacloprid (86-fold) compared with the laboratory susceptible population. While, a moderate level of resistance was observed against cypermethrin (34-fold), thiamethoxam (34-fold), nitenpyram (30-fold), chlorfenapyr (29-fold), and acetamiprid (21-fold). On the other hand, the resistance was low against bifenthrin (18-fold) after selection for five generations. A very low level of resistance against the field population of *B. tabaci*, at G1, showed that these insecticides are still effective, and thus can be used under the field conditions for the management of *B. tabaci*. However, the proper rotation of insecticides among different groups can help to reduce the development of resistance against insecticides.

## 1. Introduction

Cotton is a major cash crop, and Pakistan is the fourth largest cotton producer in the world. The vertical tap root system makes the cotton tolerant to high temperatures and drought [1]. *B. tabaci* is a major pest of cotton that damages by direct feeding, reduces seed quality through the excretion of honeydew, subsequently develops sooty mold [2], and transmits leaf curl virus [3]. *B. tabaci* was ranked 5th among the world’s top 12 most insecticide-resistant insect species [4]. Resistance develops readily in insect pests due to the unwise and indiscriminate use of insecticides [5,6]. However, the development of resistance against insecticides is a global concern. Therefore, an improved understanding of resistance could be a helpful tool for preparing insect management strategies. A laboratory selection with insecticides is one of the distinct methods used to determine the risk of insecticide resistance development.

Against both biotypes of *B. tabaci* (B biotype and Q biotype), the status of insecticides resistance development has been reported from many countries [7,8]. In Pakistan, resistance was moderate against pyrethroids, while high against cypermethrin in the field-collected B biotype of *B. tabaci* [9]. However, the population of both biotypes collected from Germany, Turkey and the UK displayed a very high resistance against bifenthrin [10]. The B biotype of *B. tabaci* also displayed very high resistance against cypermethrin and bifenthrin in Urumqi [11].

In Iran and Turkey, a low to very high resistance was observed against the neonicotinoid insecticides in the B biotype of *B. tabaci* [12]. The resistance against neonicotinoids was also very high in the field population of both biotypes of *B. tabaci* in the Jiangsu, Guangdong, Yunnan, and Zhejiang provinces of China [13]. Increased resistance levels were observed against imidacloprid and thiamethoxam [14]. However, a low to moderate level of resistance against insect growth regulators was observed in the field collected Indian strain of *B. tabaci* [15].

Continuous insecticide selection is one of the main reasons for the development of resistance in *B. tabaci* [16]. In 2011, a low level of resistance against neonicotinoids was reported in the laboratory selected *B. tabaci* population of Pakistan [17]. Whereas in 2019, a resistance ratio of up to 2461-fold against pyrethroids, and up to 2000-fold against neonicotinoids was observed in laboratory selected *B. tabaci* populations [18]. In Pakistan, the neonicotinoids were introduced in the mid-1990s. From 2000 to 2010, the resistance level was low against neonicotinoids, but due to a heavy reliance on these pesticides, the resistance increased to a high level against the field collected population in 2015. The resistance was considered none (RF ≤ 1), very low (RF = 2–10), low (RF = 11–20), moderate (RF = 21–50), high (RF = 51–100), and very high (RF > 100) [19].

Therefore, in the present study, we evaluated the effectiveness of different insecticides which are still in use to control the whitefly population. Among pyrethroids, we selected bifenthrin and cypermethrin. Meanwhile, acetamiprid, nitenpyram, imidacloprid, and thiamethoxam were selected among neonicotinoids. The efficacy of chlorfenapyr and buprofezin (IGR) was also evaluated. Furthermore, the development of resistance was monitored for five generations of whitefly. Such studies not only displayed the development of resistance against the selected insecticides, but would also be helpful to plan novel strategies to minimize or prevent resistance development in whiteflies.

## 2. Materials and Methods

### 2.1. Insect Strains

In Pakistan, the population of *B. tabaci* belongs to haplotype PCG-1 [20]. Two strains of *B. tabaci* were used for this experiment. An insecticide susceptible strain (Lab-PK) was obtained from Bahauddin Zakariya University (BZU), Multan, Pakistan, and maintained on cotton plants (var VH-305) in the entomological laboratory of the University of Agriculture Faisalabad (UAF), Faisalabad, Pakistan under a photoperiod of 16 h at of 26 ± 2 °C. Further, this insecticide susceptible strain was reared without selection with insecticides for more than 10 years [17].

Another field strain of *B. tabaci* was collected from the cotton fields of Faisalabad, Punjab, Pakistan by a battery-operated aspirator. The field collected strain was also maintained on the cotton plants (var VH-305) under the same laboratory conditions as described above.

### 2.2. Insecticides

The following commercial formulations of selected insecticides were used for the bioassay: bifenthrin 10 g a.i. l-1 (bifenthrin^®^; Anza, Pakistan), cypermethrin 10 g a.i. l-1 (cypermethrin^®^; Anza, Pakistan), acetamiprid 20 g a.i. l-1 (Rapid^®^; Anza, Pakistan), imidacloprid 20 g a.i. l-1 (imidacloprid^®^; Anza, Pakistan), thiamethoxam 25 g a.i. l-1 (Contest^®^; Anza, Pakistan), nitenpyram 10 g a.i. l-1 (Seradix^®^; Anza, Pakistan), chlorfenapyr 36 g a.i. l-1 (Kalorfen^®^; Anza, Pakistan), and buprofezin 25 g a.i. l-1 (buprofezin^®^; Anza, Pakistan) (Table 1).

### 2.3. Bioassays

Among the bioassays, the adult bioassay was followed against bifenthrin, cypermethrin, acetamiprid, imidacloprid, thiamethoxam, nitenpyram, and chlorfenapyr; the nymphal bioassay was followed against buprofezin. Fresh cotton leaves, without any exposure to insecticides, were used for all bioassays.

#### 2.3.1. Adult Bioassay

Cotton plants at two true leaf stage (20 days old) were used for the bioassay. Both leaves were immersed in insecticide solutions for 10 s with a little agitation. After that, the cotton leaves were air dried for about 1 h. Twenty-five adult whiteflies were sedated with CO_2_ and confined on the lower surface of the leaves by using a clip cage. Each insecticide was applied at five concentrations and each concentration was replicated five times, in addition to control. Furthermore, each plant was used as a single replicate and all the bioassays were conducted at controlled laboratory conditions as described above. The data of mortality was assessed after 48 h of exposure to insecticides.

#### 2.3.2. Nymphal Bioassay

A nymphal bioassay was also conducted on the whole cotton plants at the two true leaf stage. By using clip cages, 20 adult pairs of *B. tabaci* were confined on the cotton leaves for 24 h, at conditions as described above. Later, the adults were removed and the seedlings were placed in the growth chambers for 12 days until the nymphs reached their second instars. The nymphs were counted by using a stereo-microscope, and the leaves (with 15–25 nymphs) were dipped in the insecticide solutions for 5 s. Five concentrations of each insecticide were used, and each concentration was replicated five times, in addition to a control. Each cotton plant was used as a single replicate, and data were recorded after 22 days of egg-laying by counting the number of emerging adults [21].

### 2.4. Selection with Insecticides

The field-collected population of whiteflies was subjected to a selection against different insecticides for five generations. For bifenthrin, cypermethrin acetamiprid, imidacloprid, thiamethoxam, nitenpyram, and chlorfenapyr, the selection was done by exposing the adults with different concentrations (0.5, 1, 10, 160, and 300 ul L^−1^ from G1 to G5) of insecticides. For buprofezin, the selection was done by exposing the 2nd instar nymphs to different concentrations (0.5, 1, 10, 160, and 300 ul L^−1^ from G1 to G5).

For the adult bioassay, the number of adults selected per generation ranged from 1500–3500, over five generations of selection. However, for the nymphal bioassay, the number of nymphs selected per generation ranged from 800–2500, over five generations of selection.

### 2.5. Statistical Analysis

At the end of each experiment, the LC_50_ value of each generation was calculated, and the overall development of insecticide resistance levels for up to five generations were presented and compared statistically in context to the susceptible strain. Furthermore, Abbott’s formula was used, and data were corrected for the control mortality, where necessary. LC_50_ values and their 95% FLs were obtained by probit analysis by using POLO computer-based statistical software (POLO, LeOra software, Menlo Park, CA, USA). The resistance ratio against each insecticide in different generations was also calculated by dividing the LC_50_ of the insecticide-selected population to the LC_50_ of the insecticide-susceptible population. The graphs of mortality were prepared by using GraphPad Prism. Due to the inherent variability of bioassays, pairwise comparisons of LC_50_ values were performed at the 5% significance level (where individual 95% FLs for two treatments do not overlap).

## 3. Results

### 3.1. Toxicity of the Insecticides against the Lab-PK and Field Population

For the Lab-PK population, toxicities of acetamiprid, imidacloprid, thiamethoxam, nitenpyram, chlorfenapyr, and buprofezin were notably higher as compared to bifenthrin and cypermethrin (Table 2). However, buprofezin was the most toxic insecticide against the Lab-PK, whereas bifenthrin was the least toxic insecticide. The slope of the Lab-PK population with cypermethrin was the shallowest, suggesting a heterogeneous response. However, the steeper slope for bifenthrin showed a homogenous response (Table 2).

Compared with the Lab-PK, the toxicities of all tested insecticides were significantly lower (*p* < 0.05) in the field population at G1, suggesting a homogenous response in the field population against these insecticides (Table 2).

### 3.2. Response to Selection with Insecticides

**Figure 1:**
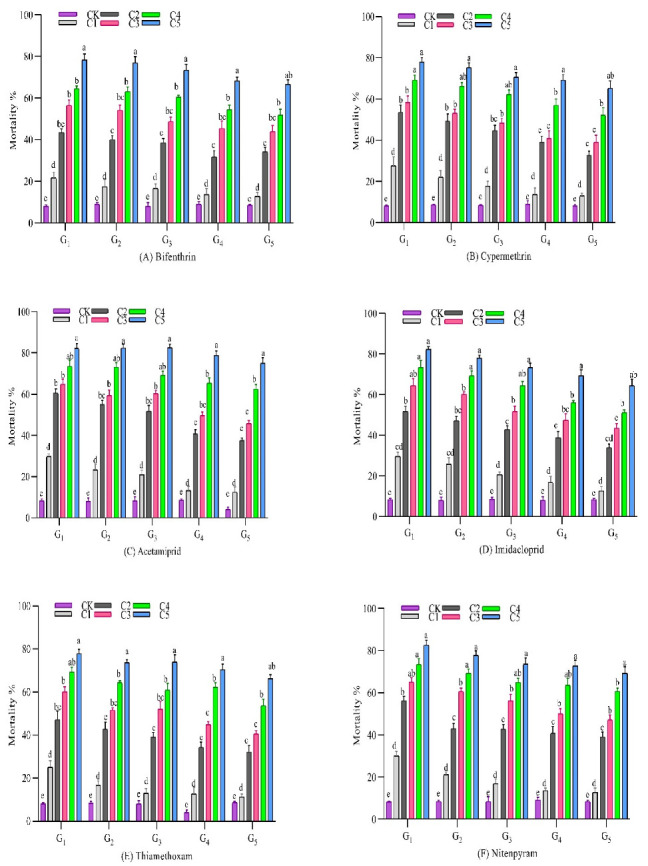

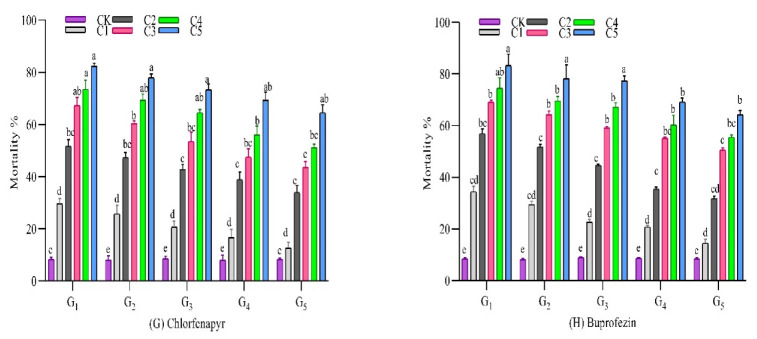
Mortality percentage of *B. tabaci* to five concentrations of eight different insecticides (**A**–**H**), at five generations. (G = generation, C = concentration, CK = control). Means followed by the same letter (a–e) above vertical bars are not significantly different at 5% level of probability.

The field collected whitefly population was selected with eight different insecticides for five generations. However, each insecticide was applied at five concentrations (0.5, 1, 10, 160 and 300 µL/L) in each generation. Furthermore, a decrease in the mortality % age was observed among the successive generations, indicating the development of resistance (Figure 1).

#### 3.2.1. Bifenthrin

Resistance was at a very low level (*p* < 0.05) in the field collected population of whiteflies. A selection for five generations changed the resistance to a low level (*p* < 0.05) with the resistance ratio of 18-fold as compared to the laboratory susceptible population (Table 3).

#### 3.2.2. Cypermethrin

The exposure of whiteflies to cypermethrin showed a gradual rise in resistance over five generations. The resistance was very low at G1 (*p* < 0.05), and low at G2 (5-fold, *p* < 0.05). However, as generations progressed, the resistance reached a moderate level at G4 (23-fold, *p* < 0.05) and stabilized at G5 (34-fold, *p* < 0.05).

#### 3.2.3. Acetamiprid

The resistance against acetamiprid rose from a very low level (1-fold, *p* < 0.05) at G1 to a moderate level (21-fold, *p* < 0.05) at G5. These results indicated that the selection with acetamiprid significantly increased the resistance development in *B. tabaci*, after selection for five generations.

#### 3.2.4. Imidacloprid

A range in the change of resistance was observed during selection with the imidacloprid for five generations. Resistance was very low (4-fold, *p* < 0.05) in the field-collected whitefly population. Further selection increased the resistance to a low level at G3 (18-fold, *p* < 0.05), moderate level at G4 (41-fold, *p* < 0.05), and to a high level (85-fold, *p* < 0.05) at G5, as compared to the laboratory susceptible population (Figure 1).

#### 3.2.5. Thiamethoxam

The rate of resistance development against the thiamethoxam-selected population of *B. tabaci* was found to be slow. The results showed that after selection for four generations, the resistance reached a low level (19-fold, *p* < 0.05). However, it increased to a moderate level at G5 (34-fold, *p* < 0.05), as compared to the laboratory-susceptible population.

#### 3.2.6. Nitenpyram

Results showed a very low level of resistance development (3-fold, *p* < 0.05) against nitenpyram in the field-collected population of whitefly. Resistance rose from a low level in G3 (14-fold, *p* < 0.05) to a moderate level in G5 (30-fold, *p* < 0.05), as compared to the laboratory susceptible-population.

#### 3.2.7. Chlorfenapyr

The rate of resistance development against the chlorfenapyr selected population of *B. tabaci* was also found to be slow. The results showed that after selection for four generations, the resistance rose to a low level (15-fold, *p* < 0.05). However, it increased to a moderate level at G5 (30-fold, *p* < 0.05) as compared to the laboratory-susceptible population.

#### 3.2.8. Buprofezin

The results indicated that selection with buprofezin significantly increased the resistance development in *B. tabaci*. The resistance was found to be at very low level in the field-collected whitefly population (3-fold. *p* < 0.05). However, continuous selection for up to five generations increased the resistance development to a very high level at G5 (127-fold, *p* < 0.05), as compared to the laboratory-susceptible population (Table 3).

## 4. Discussion

Insect pests directly affect the production of agriculture by causing damage to the food commodity. These insects cause damage to the agricultural crops in two major ways. In the first way, they directly cause damage by feeding on the agricultural commodity. In the second case, they are responsible for transmitting bacterial, viral, and fungal infection to field crops. So, in the second case, the insect itself is not responsible for damage; it is instead the microbial infection the insect carries that results in damage to agricultural crops. Regardless of the adverse effects produced by the insecticides, chemical control is still the first line of defense to control the insect pests. In Pakistan, the farmers usually practice up to eight sprays of neonicotinoids or IGRs, either combined or singly [22]. To avoid resistance development in the insect pests, the regular monitoring of the field crops for the pest spread is a basic step. Due to the reports of poor control of the *B. tabaci*, the present study was conducted to find out the development of resistance against eight different insecticides in the population of *B. tabaci*.

Our findings showed that the highest resistance development was observed in the population selected with buprofezin for five generations (126.91-fold), followed by imidacloprid (85.94-fold), thiamethoxam (34.21-fold), cypermethrin (33.65-fold), nitenpyram (29.93-fold), chlorfenapyr (29.46-fold), acetamiprid (21.10-fold), and bifenthrin (17.51-fold), respectively, as compared to the laboratory-susceptible population. The overall insecticide resistance development against buprofezin was found to be very high, high against imidacloprid, and low against bifenthrin. Such results were explained by the increased activity of Glutathione S-transferases, which was responsible for the resistance development in the Q biotype of *B. tabaci* against neonicotinoids [23]. However, many studies also reported that the insecticide selection increased resistance development in the B biotype of *B. tabaci*. For example, a 490-fold increase in resistance was observed after selection of *B. tabaci* (B biotype) for 30 generations with the imidacloprid [24]. Meanwhile, moderate to high resistance was observed, against the thiamethoxam and acetamiprid after selection of *B. tabaci* for five generations [25]. One other reason for such an increase in the resistance development could be the extensive use of insect growth regulators and neonicotinoids. Since their recent development, these insecticides are used more frequently to control the insect pests in the field, so this might be a reason that the resistance developed more readily against these insecticides. On the other hand, a little reliance on bifenthrin was responsible for its low resistance in *B. tabaci*. Another study also reported such findings, and an up to 14-fold increase in resistance to bifenthrin was reported in the whitefly population after a selection for four generations [26]. However, resistance increased to a high level in the Q biotype of *B. tabaci* after a selection with imidacloprid for nine generations [27]. On the other hand, resistance increased from low to a moderate level in both biotypes of whiteflies after selection for 26 generations with acetamiprid [28]. Insecticide selection also increased resistance development from a moderate to high level against cypermethrin [29]. However, moderate resistance was reported after selection with chlorfenapyr [30]. Furthermore, the insecticide resistance against neonicotinoids and IGRs has also been reported in other insect pests such as *Aphis gossyp*, *Frankliniella fusca*, *Spodoptera litura*, *Spodoptera littoralis*, and *Spodoptera frugiperda* [31,32,33,34].

At the first generation, the whitefly population displayed a very low level of resistance against bifenthrin (2.88), cypermethrin (2.44), acetamiprid (1.37), nitenpyram (2.71), imidacloprid (3.83), thiamethoxam (3.31), chlorfenapyr (2.62), and buprofezin (3.14), respectively. Thus, a low level of resistance against the pyrethroids, neonicotinoids, chlorfenapyr, and insect growth regulator in the field-population of whiteflies indicated that these insecticides are still effective against *B. tabaci* [35,36,37]. Different levels of resistance against different insecticides, at same level of exposure, may be due to several factors, including their mode of action and the heavy reliance on these insecticides. For example, if an insecticide is applied more frequently, then there are more chances of resistance development as compared to another insecticide from a different group.

To effectively manage the resistance-related problems, awareness to the stability of resistance is very important. If an insecticide is found to be unstable, then that specific insecticide can be removed from the spraying schedule to minimize the resistance development. Many studies have also proved that such management tactics helped in overcoming resistance development, and the resistance was also found to be unstable in the absence of a selection with insecticides [38]. The RR_50_ value for imidacloprid dropped from 60 to four just after six generations, without selection with insecticides [39]. Age-specific expressions of resistance development are also very important, as they provide valuable information regarding the status of insect pests. Furthermore, a proper understanding of the insect endocrine system also helps to better understand the insect reproductive system, which in turn allows the development of new strategies to control insect pest [40]. If an immature stage is more sensitive, then such a stage can be targeted to not only control the insect pests, but also to keep the resistance development under control [21]. The apprehension of genetic, inheritance pattern, transcriptional regulatory mechanisms and other approaches is also important to have a better understanding on the development of resistance at molecular level [41,42].

## 5. Conclusions

The results of the present study showed that the selected insecticides are effective against the field-collected whitefly population [17], and thus can be used under the field conditions for the management of *B. tabaci*. The current study also displayed a gradual increase in resistance development over five generations, suggesting that the continuous exposure of a pest to a particular group of insecticide can result in the development of resistance. Therefore, it is concluded that even though the field population was susceptible at G1, there is a greater risk of resistance development if successive generations are exposed to a peculiar group of insecticides. Thus, it is recommended that a rotation of insecticides should be considered as a key part of control strategies to minimize the likelihood of resistance development.

The current study provides crucial information, implying that an insecticide rotation program should be considered while designing pest management strategies. Furthermore, this information lays a foundation for further studies in understanding the complex mechanisms involved in resistance development.

## Figures and Tables

**Table 1:** Trade name, active ingredients (AI), formulation, Field rate and IRAC main group of selected insecticides.

Trade Name	Active Ingredient	Formulation	Field Rate Acre^−1^	IRAC Main Group
Rapid	Acetamiprid	20 SL	250 mL	4A
Kalorfen	Chlorfenapyr	36 SC	225 mL	13
Imidacloprid	Imidacloprid	20 SL	250 mL	4A
Contest	Thiamethoxam	25 SC	200 mL	4A
Seradix	Nitenpyram	10 SL	125 mL	4A
Bifenthrin	Bifenthrin	10 EC	250 mL	3A
Cypermethrin	Cypermethrin	10 EC	250 mL	3A
Buprofezin	Buprofezin	25 EC	450 mL	16

IRAC = Insecticide resistance action committee.

**Table 2:** The response of laboratory susceptible (Lab-PK) and field population of *B. tabaci* against different insecticides.

Population	Insecticides	LC_50_ (95% FL)(ug a.i. mL^−1^)	Slope (±SE)	Probit Fit Line			RR^a^
				χ^2^	*df*	*p*	
Lab-PK	Bifenthrin	3.19 (1.05–7.01)	0.17 ± 0.02	8.43	5	0.038	-
Lab-PK	Cypermethrin	1.91 (0.54–4.44)	0.16 ± 0.02	12.63	5	0.006	-
Lab-PK	Acetamiprid	1.08 (0.41–2.18)	0.22 ± 0.02	17.20	5	0.001	-
Lab-PK	Imidacloprid	0.65 (0.15–1.66)	0.17 ± 0.02	8.54	5	0.036	-
Lab-PK	Thiamethoxam	1.47 (0.57–2.95)	0.21 ± 0.02	11.72	5	0.008	-
Lab-PK	Nitenpyram	0.87 (0.33–1.75)	0.22 ± 0.02	9.99	5	0.019	-
Lab-PK	Chlorfenapyr	0.52 (0.12–1.34)	0.17 ± 0.02	8.12	5	0.044	-
Lab-PK	Buprofezin	0.35 (0.08–0.93)	0.17 ± 0.02	8.13	5	0.043	-
Field (G1)	Bifenthrin	9.19 (4.32–17.16)	0.20 ± 0.03	10.54	5	0.014	3
Field (G1)	Cypermethrin	4.66 (1.80–9.50)	0.18 ± 0.02	11.13	5	0.011	2
Field (G1)	Acetamiprid	1.48 (0.43–3.40)	0.17 ± 0.02	16.08	5	0.001	1
Field (G1)	Imidacloprid	2.49 (0.91–5.12)	0.19 ± 0.02	9.07	5	0.028	4
Field (G1)	Thiamethoxam	4.86 (1.96- 9.68)	0.19 ± 0.02	9.42	5	0.024	3
Field (G1)	Nitenpyram	1.89 (0.61- 4.14)	0.18 ± 0.02	12.74	5	0.005	3
Field (G1)	Chlorfenapyr	1.36 (0.37–3.24)	0.16 ± 0.02	8.25	5	0.041	3
Field (G1)	Buprofezin	1.10 (0.27–2.70)	0.16 ± 0.02	10.48	5	0.015	3

RR^a^ = Resistance ratio calculated as LC_50_ of field population/LC_50_ of Lab-PK.

**Table 3:** The response of *B. tabaci* to different insecticides after five generations of selection under the laboratory conditions.

Population	Insecticides	LC_50_ (95% FL) (ug a.i. mL^−1^)	Slope (±SE)	Probit Fit Line			RR^a^
				χ^2^	*df*	*p*	
Bifenthrin-SEL	Bifenthrin	55.86 (28.48–121.03)	0.21 ± 0.03	12.29	5	0.006	18
Cypermethrin-SEL	Cypermethrin	64.27 (33.67–136.34)	0.22 ± 0.03	9.77	5	0.021	34
Acetamiprid-SEL	Acetamiprid	22.74 (12.76–39.06)	0.25 ± 0.03	13.33	5	0.004	21
Imidacloprid-SEL	Imidacloprid	55.86 (28.48–121.03)	0.21 ± 0.03	12.29	5	0.006	86
Thiamethoxam-SEL	Thiamethoxam	50.29 (27.04–99.44)	0.22 ± 0.03	10.01	5	0.018	34
Nitenpyram-SEL	Nitenpyram	26.04 (13.86–48.04)	0.22 ± 0.03	14.69	5	0.002	30
Chlorfenapyr-SEL	Chlorfenapyr	15.32 (6.83–31.61)	0.18 ± 0.03	19.04	5	0.000	29
Buprofezin-SEL	Buprofezin	44.42 (22.85–91.60)	0.21 ± 0.03	11.76	5	0.008	127

RR^a^ = Resistance ratio, calculated as (LC_50_ of field population)/(LC_50_ of Lab-PK).

## Data Availability

Not applicable.
